# Improving Public Health and Governance in COVID-19 Response: A Strategic Public Procurement Perspective

**DOI:** 10.3389/fpubh.2022.897731

**Published:** 2022-05-30

**Authors:** Ran Yan, Fuguo Cao

**Affiliations:** ^1^School of Public Finance and Taxation, Central University of Finance and Economics, Beijing, China; ^2^Fanli Business School, Nanyang Institute of Technology, Nanyang, China

**Keywords:** emergency procurement, strategic procurement, COVID-19, public health, governance

## Abstract

Good governance is the basis of dealing with major emergencies and protecting public health. It has become a major issue of the central government to construct a scientific procurement and supply system of emergency supplies. This article constructs the analytical framework of strategic procurement and expounds the realization mechanism of strategic procurement under an emergency situation to reflect China's procurement practice in COVID-19 response and improve public health and governance. Using case study, semi-structured interviews, and the Nvivo text analysis, this study found that emphasizing the strategic function of securing the public health,the top status of MSG, cross-sector procurement team, strong procurement and supply integration, comprehensive and in-depth procurement synergy mechanism are the successful experiences of China's emergency procurement. However, due to the temporary nature of the emergency procurement mechanism, strategic procurement planning, procurement management specialization, and procurement supply integration still need to be improved. The findings of this study further suggest that to improve public health and governance, it is pivotal to reconstruct the government procurement law to make it compatible with the emergency procurement and transform the government procurement system into a strategic procurement in a consistent and coherent way.

## Introduction

Rapid procurement and efficient allocation of emergency supplies are crucial to the government's response to emergencies, which can ensure that public institutions hedge and mitigate social risks (1–3). Under ordinary emergencies, the government can quickly activate emergency plans (4–6), use reserves (7, 8), and current procurement to meet the material needs (9–11). However, in the context of major emergencies, such as COVID-19, the public sector's demand for emergency supplies is extremely urgent and shows explosive growth (12, 13), and the supply chain is fragile (14–16), which makes the market of emergency supplies in a shortage (17, 18) and brings unprecedented challenges to public procurement management (19–21). In response to the COVID-19 outbreak, China has adhered to the common value of prioritizing people's life and health and implemented an unconventional centralized procurement and supply model of emergency supplies through the joint prevention and control mechanism, so as to ensure the matching of demand and supply of emergency supplies in a relatively short period of time.

However, China's government procurement law excludes emergency procurement from its scope of application. Although the temporary emergency procurement mechanism has achieved good results, it lacks connection with the conventional public procurement system. Although the extreme emergency situation similar to the initial outbreak of COVID-19 has passed, the risk of major emergencies is inevitable. Therefore, we still need to reflect on the public procurement system and the practice to deal with COVID-19 to build a regular and more scientific emergency procurement system, then secure public health.

After the outbreak of COVID-19, the representative viewpoints on emergency procurement advocated the improvement of relevant procurement procedures and procurement methods (22, 23), building a whole chain of emergency procurement supervision and management system (24), establishing a supply chain risk management and synergy mechanism, and optimizing the decision-making capacity of reserves (25, 26). From the perspective of emergency management, other scholars pointed out that active anti-epidemic willingness, government-centered resource mobilization and unified scheduling, and the coordination and cooperation among multiple sectors (27–29) are China's successful experiences in dealing with emergency material support. However, the existing literature lacks a systematic reflection on China's emergency procurement against COVID-19.

The practice of China's emergency procurement in response to COVID-19 pandemic is largely in line with the theory of strategic procurement. We can examine it from the perspective of strategic procurement because procurement can ensure that organizations have enough resources and capabilities (30) to achieve their core strategic goals under the theory of strategic procurement. Strategic procurement attaches great importance to the integration of supply chain, which can widely mobilize the resources and capabilities of the whole society and reduce social uncertainty. Vecchi et al. (31) have used this theory to analyze the public procurement in the United States and Italy in response to COVID-19. However, they did not respond to the mechanism of strategic procurement, nor did they systematically examine cases. Based on the perspective of strategic procurement, this study summarizes the dimensions of strategic procurement and analyzes the realization mechanism of strategic procurement in emergency situations. Combined with the case study and text analysis of procurement in X Province and Y City in response to the epidemic, this study examines the application of strategic procurement in China's emergency procurement management system.

The possible marginal contributions of this article are as follows: First, it constructs an analytical framework for strategic procurement in emergency situations and explains its realization mechanism, thereby deepening the explanatory power of the effectiveness of the centralized procurement and supply system adopted by China in response to COVID-19. Second, it provides empirical evidence for the improvement of the emergency procurement system through an in-depth case study. The third is to put forward suggestions for the reconstruction of China's public procurement system, transforming China's fruitful experience into a normalized emergency procurement system.

## Theoretical Perspective of Strategic Procurement

Emergency procurement is the procurement by the public sector to meet the need for emergency supplies arising from emergencies, such as natural or man-made disasters and emergency military actions (32). The time, place, degree of harm, and demand for emergency materials are highly uncertain, and the production and supply of emergency supplies are also uncertain due to supply chain risks (33). Especially in the case of major emergencies, due to the short-term surge in demand, the market for emergency supplies presents typical characteristics of a shortage market, such as buyers' competitive purchases, rapid price increases, and poor quality (34). Therefore, the high complexity of emergency procurement management correspondingly brings new challenges to the public sector (35, 36). The public sector that is more accustomed to a competitive market with sufficient supply, the main content of its procurement management, has also shifted from focusing on competitive procurement to ensuring supply, and the procurement method has also changed from a competitive mechanism to a more time-sensitive and flexible procurement. The breadth and depth of coordination between or inside different sectors have also increased unprecedentedly. Strategic procurement is beneficial to meet the challenges of emergency procurement management.

### Applicability of Strategic Procurement in Emergency Procurement Management

The strategic procurement theory originated from the field of business administration, which is the process of planning, implementing, evaluating, and controlling procurement decisions, so as to make procurement activities serve the core goals of the organization (37). As resources are the core competence of allocation, development and integration of resources have strategic function (38–40), and they can be obtained through procurement, so procurement also has strategic function. Furthermore, procurement has gradually transformed from a simple market transaction activity to a strategic activity that affects the organization's sustainable competitive advantage and supports the organization's value system (41), then forms a strategic management function that is different from the traditional procurement function. Under the perspective of strategic procurement, the connotation of procurement has also been greatly expanded, including procurement, supply management, supplier development and innovation, and partnership (42).

Strategic procurement is increasingly being used to study how the public sector uses resources and capabilities to provide public value to its stakeholders (43). Strategic procurement requires the public sector to value the strategic role of procurement and public-private partnerships (31), transform procurement into a more strategic governance process, and proactively plan procurement activities to align with the public organizations' core values and consistent with the long-term strategy, finally achieve the policy function of public procurement (44). In practice, public procurement is also increasingly involved in organizational strategy (45, 46). For example, the WHO and the World Bank (WB) use strategic procurement as a way to improve the health system performance and achieve a universal health coverage; the UK uses strategic procurement to ensure the right public services are delivered in the right way (47). For the public sector, the difference between strategic procurement and traditional procurement is shown in Table 1.

**Table 1:** Differences between strategic procurement and traditional procurement.

	**Traditional procurement**	**Strategic procurement**
Procurement function	Passively meet short-term needs of goods and services	Proactively plan procurement activities to serve the core strategic objectives of the organization
Procurement and supply management	Procurement costs, compliance and prevention of corruption	Promote innovation and risk management through sectoral collaboration to achieve social policy objectives
Supplier relationship	Trading relationship	Long-term partnership, integrating internal and external resources and capabilities through procurement and supply
Status of Procurement Department	Compared with other departments, the status is not high enough	Have the function of strategy management

**Figure 1:**
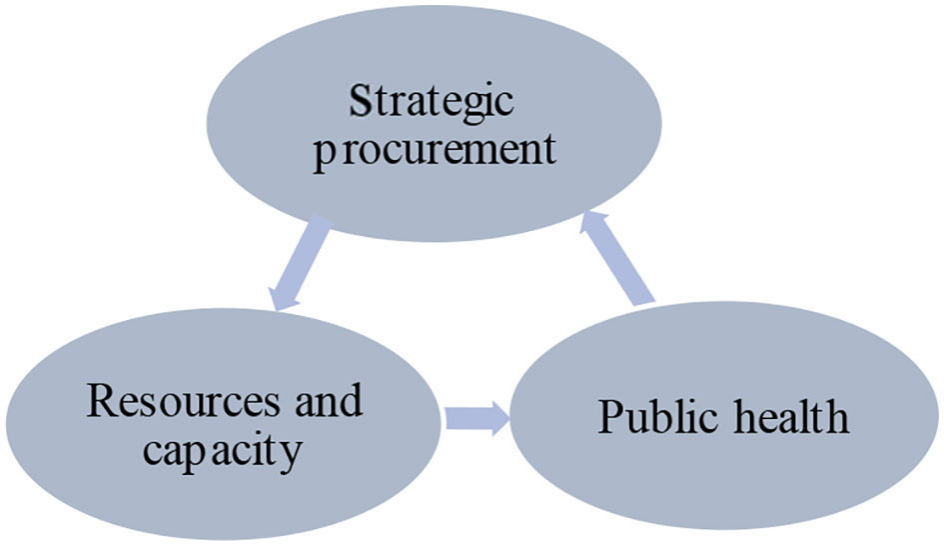
The relationship between strategic procurement and public health.

Under the emergency situation, the public sector, similar with enterprises, faces the uncertainty of internal and external environments and needs to build long-term partnerships with suppliers to hedge risks. Resources and capabilities are also unique assets that determine the public sector's ability to respond to emergencies. Procurement of emergency resources is also a strategic procurement process that aligns and synchronizes internal needs with external resources (48), removes barriers to resource mobilization during a disaster (49), and ultimately provides resources and capacity for emergency management. The core of emergency management is public-risk management. Emergency procurement has the strategic function of defusing public risks by providing the material basis necessary for emergency management (50, 51). Similarly, as is shown in Figure 1, strategic procurement is beneficial for public health, and public health needs strategic procurement.

### Perspective of Strategic Procurement

#### Dimension of Strategic Procurement

The strategic function of procurement is the starting point of strategic procurement. An organization's procurement function is strategic, non-strategic, or somewhere in between depending on the degree to which strategic procurement is achieved (52) and can be measured by the strategic procurement dimension. Drawing on the related research results (24–27, 31, 33, 44, 47, 51–62), this article summarizes the key factors of strategic procurement (Table 2), including motivation, dimensions, and function. Dimension is the core of strategic procurement. It includes six mutually supporting aspects: strategic procurement objectives, strategic procurement planning, status of procurement department's, specialization of procurement management, procurement supply integration, and procurement synergy mechanism.

**Table 2:** Key factors of strategic procurement under the emergency.

**Level 1 factors**	**Level 2 factors**	**References**
Motivation of strategic procurement	Needs of emergency management	(31, 54)
	Market uncertainty	(33, 37, 48, 52, 53)
	Management capacity	(35, 36)
Dimension of strategic procurement	Strategic procurement planning	(30, 37, 44, 53, 55, 56)
	Status of procurement department	(37, 53, 57)
	Specialization of procurement management	(24, 36, 37, 57–59)
	Procurement and supply integration	(25, 31, 33, 53, 60, 61)
	Procurement synergy mechanism	(31, 48, 61, 62)
Strategic function	Resolving public risks	(31, 50, 53)

First of all, strategic procurement requires that the procurement objectives be consistent with the strategic objectives of the organization, so that procurement can support the organizational value (37, 55). The government's response to emergencies aims to safeguard the public health and property, national security, and social stability. Emergency procurement serves the organizational goal of resolving public risks and ensuring public security. Secondly, strategic procurement requires the formulation of strategic procurement plan based on the organization's strategic goals. Procurement strategic planning is the essential preparation for emergencies (63). Thirdly, the status of procurement department refers to the status of procurement department in the organization, representing the importance attached by the top management to procurement (37, 53). The higher the status of the procurement department, the easier it is to realize the strategic function of procurement. Fourthly, the specialization of procurement management requires the procurement department to have professional functions and procurement personnel to have professional skills, which can meet the needs of strategic procurement (57–64). The specialization of procurement is the skill guarantee for the implementation of strategic procurement. Fifthly, procurement and supply integration refer to establishing partnerships with suppliers through procurement and effectively integrating internal and external resources to achieve organizational goals (31, 60, 61). Procurement and supply integration are the key to maintaining supply chain resiliency, fostering organizational integration innovation, and implementing risk management. Sixthly, the procurement synergy mechanism is a unified command, coordinated and orderly working mechanism in the implementation of strategic procurement. Strategic procurement is a systematic and complex strategic activity, which requires synergy between inside and outside powers of the organization through the procurement synergy mechanism (61). The synergy mechanism in emergency procurement is mainly manifested as the linkage and synergy mechanism between different departments and organizations in the process of procurement implementation.

#### Realization Mechanism of Strategic Procurement Under Emergency Situation

**Figure 2:**
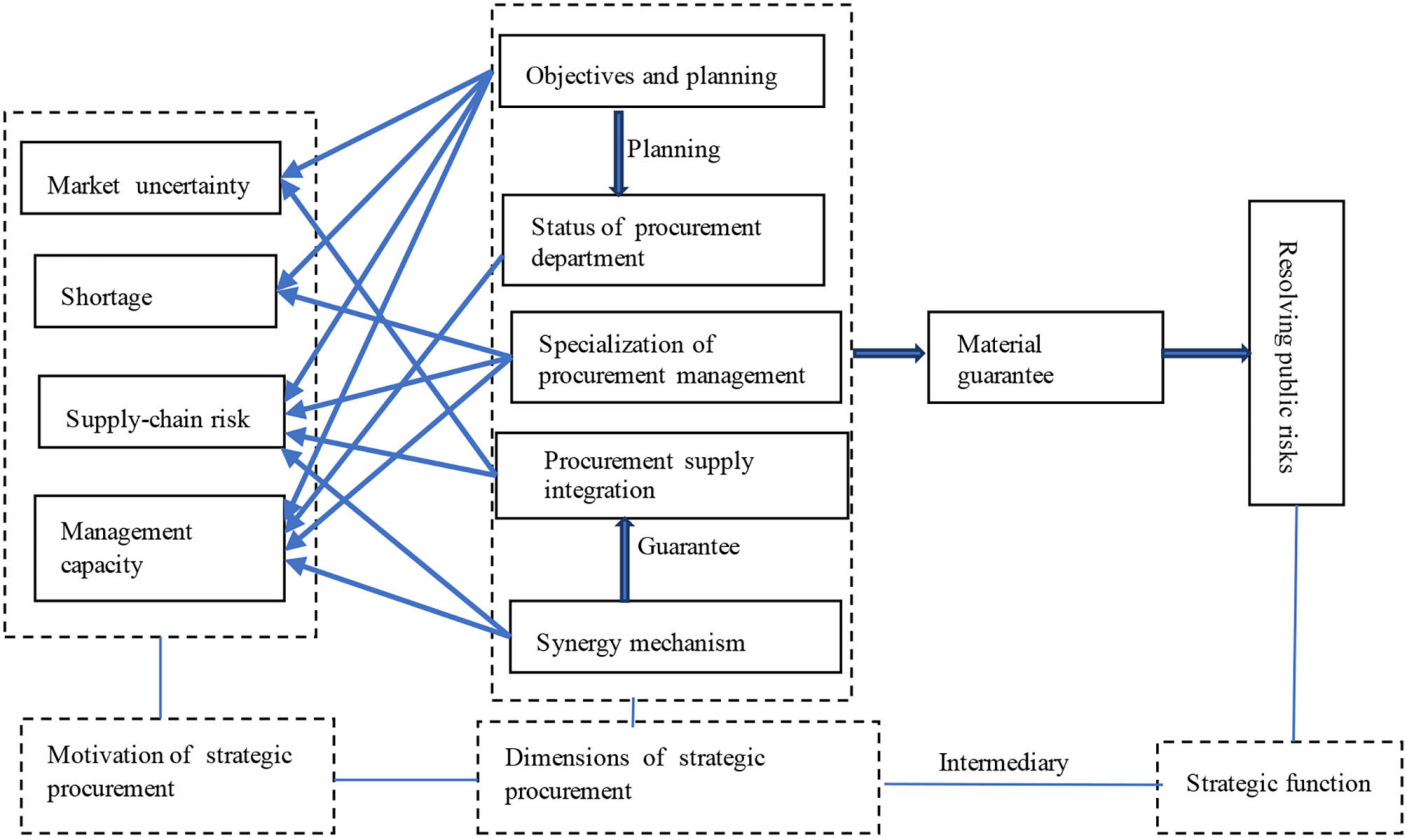
The realization mechanism of strategic procurement under emergency situation.

Combining the challenges faced by emergency procurement and the dimensions of strategic procurement, this study summarizes the realization mechanism of strategic procurement in emergency procurement management as shown in Figure 2. First, from the main line of logic, the strategic procurement dimension can specifically solve the challenges of procurement management in emergency situations, ultimately provide material support for emergency management, and realize the strategic function of resolving public risks. Second, challenges faced by emergency procurement are actually the motivation of strategic procurement, which provides sufficient basis for the implementation of strategic procurement, and is also the necessity of implementation of strategic procurement. Third, the dimensions of strategic procurement solve the problem of how to implement the strategic procurement. Strategic objectives and planning of procurement are the strategic preparation of emergency procurement. The status of procurement department, the specialization of procurement management, and the integration of procurement and supply directly determine whether strategic procurement is implemented or not. The procurement synergy mechanism is an important guarantee for the implementation of strategic procurement. Fourth, emergency procurement achieves the strategic goal of maintaining public security through the guarantee of emergency supplies. Emergency supply guarantee plays an intermediary role because it reduces social uncertainty and improves the level of resources and capacity for emergency management.

## Research Methods

### Case Study

This article mainly adopts the case study method. The case study method is a crucial way to examine the nature of a social event and study the complex context of different variables. It is also a suitable way to study how the government reacts to the COVID-19 to protect the public health from the perspective of strategic procurement. The case selected is X Province and Y City (as a whole) in China. Since China runs a unitary governance system, the lower-level government is under the leadership of the higher-level government, and the perspective of province and city is conducive to a comprehensive understanding of China's epidemic procurement management. Both X Province and Y City are with large population and high population density, and adjacent to Hubei Province, accordingly with high risk of public health. Province X belongs to provinces with severe epidemics except for Hubei Province, while City Y belongs to areas with severe epidemics in Province X. The first confirmed case of COVID-19 was detected in X Province and Y City on 21 January and 24 January 2020, respectively. As of 21 November 2020, 1,288 people had been diagnosed in X Province and 156 in Y City^1^. Province X is an important production base of sanitary materials in China. Several enterprises have been included in the national emergency medical supply allocation system, and have undertaken the designated assistance tasks for epidemic prevention materials in Hubei, Xinjiang, Ningxia, Inner Mongolia, and other provinces. However, the medical industry of Y City is weak, and it is the net inflow party of emergency medical supplies. Therefore, the procurement cases of epidemic prevention materials in X Province and Y City are highly representative. The case information of this study mainly comes from three ways: semi-structured interview records with interviewees, written materials provided by interviewees, and relevant policy documents and information collected by authors through other channels.

### Data Collection

#### Interview Materials

In this study, semi-structured interview was used to conduct face-to-face interviews with the staff involved in epidemic procurement management in X Province and Y City. The interview time of each interviewee was about 1 h. The authors prepared the interview outline for each interviewee before the interview and flexibly adjusted the interview content according to the interviewee's job characteristics and information provided during the interview. The authors also avoided disclosing the research intension. According to the dimensions of strategic procurement, the interviews mainly focused on the shortage and strategic planning of emergency supplies, procurement and supply integration, procurement synergy mechanism, the status and role of the procurement departments, and the implementation and supervision of emergency procurement, and also paid attention to the problems and suggestions found in the work of the interviewees. After the interview, the author sorted out the interview results one by one according to the interview notes and records and agreed with the interviewees on anonymous processing and data use.

China's emergency procurement management is under the responsibility of the Material Support Group (MSG) of the COVID-19 Joint Prevention and Control Headquarters (JPCH). The MSG mainly include the Bureau of Industry and Information Technology (BIIT), the Health Commission (HC), the Bureau of Finance (BF), the Auditing Bureau (AB), and the Bureau of Civil Affairs (BCA). The main demanders of emergency medical supplies are the designated hospitals, and the main suppliers are the key production enterprises. Provincial Reserve Enterprises (PRE) play the role of procurement, storing, and dispatching. Therefore, this research focuses on the interviews with 14 staff representatives from the above-mentioned organizations who participate in the procurement and supply of emergency supplies. Taking into account the representativeness of the selected cases, the authors also interviewed a staff member of the MST in L City of G Province. The detailed information of interviewees is shown in Table 3.

**Table 3:** Information table of interviewees.

**Serial number**	**Department**	**Position in MSG**
A11	BIIT of X Province	Leader of provincial MSG
A12	BIIT of X Province	Liaison of provincial MSG
A13	PRE of X Province	Helping the government procurement, reserve, and deploy medical supplies
B11	HC of Y City	Leader of MSG in N city
B12	BF of Y City	Staff member of MSG in N city
B13	BF of Y City	In charge of the special funds for fighting the epidemic
B14	AB of Y City	In charge of the special audit of epidemic procurement
B15	BCA of Y City	Staff member of MSG in N city
C11	Hospital 1 of Y City	Staff member of MSG, In charge of hospital procurement
C12	Hospital 2 of Y City	Hospital procurement
C13	Hospital 2 of Y City	In charge of the hospital special funds for fighting the epidemic
C14	Hospital 2 of Y City	Hospital procurement
D11	Enterprises 2	Ensuring disinfect alcohol supply
B21	BIIT of L City, G Province	Staff member of MSG in L city

#### Policy Documents

**Figure 3:**
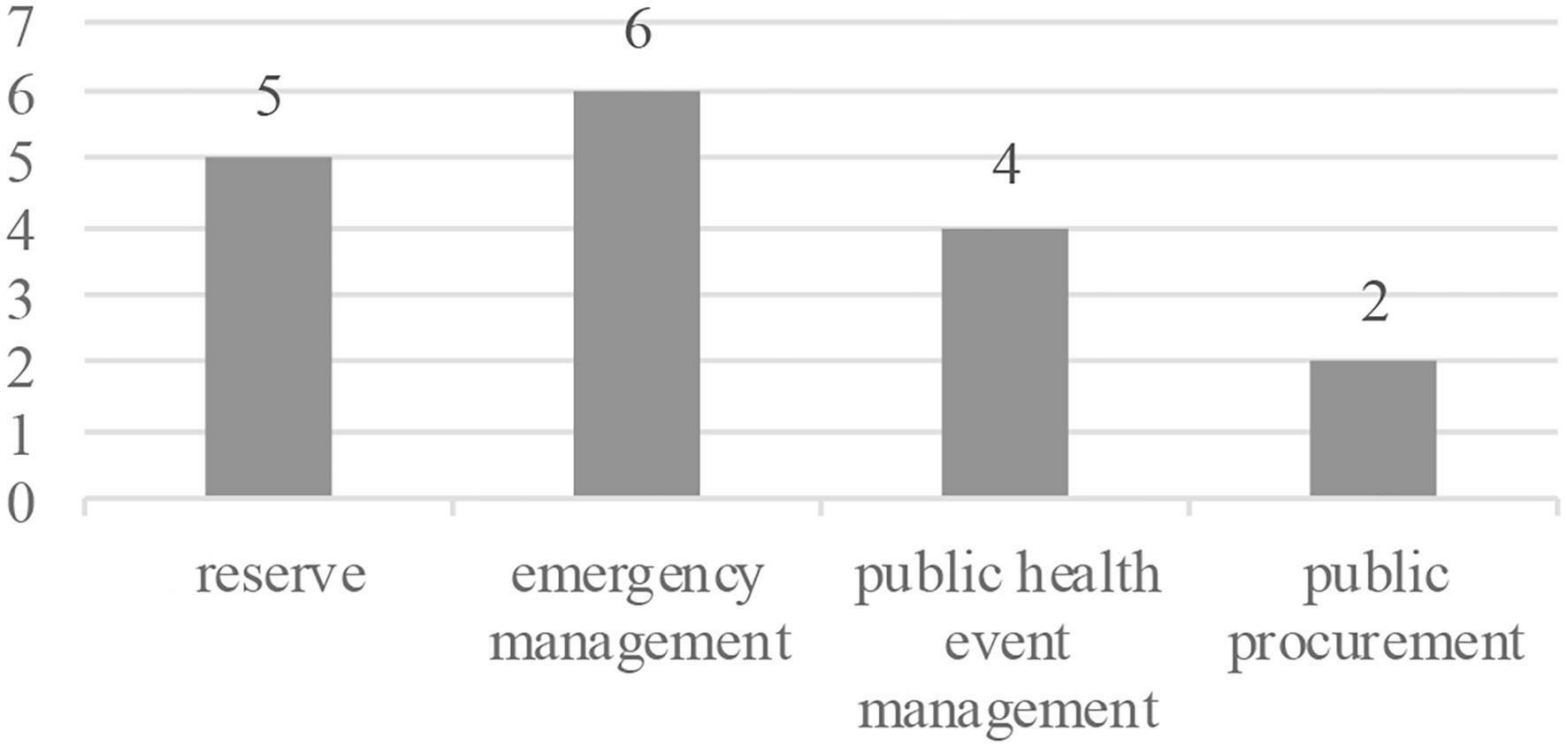
Documents before COVID-19.

**Figure 4:**
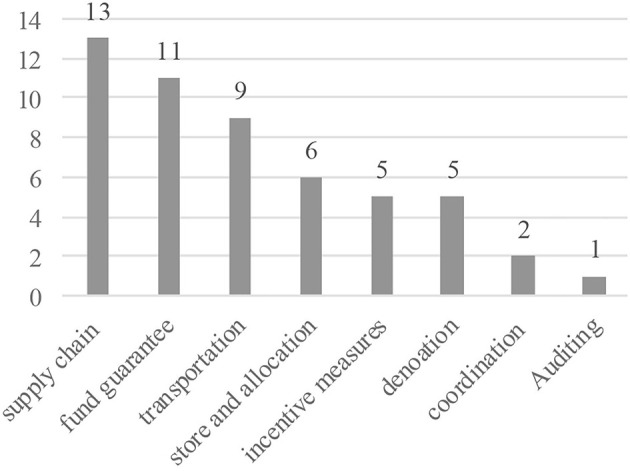
Documents after COVID-19.

To supplement the survey and interview, 69 policy documents related to emergency supply support at national, provincial, and municipal levels were collected in this study, among which 32 policy documents at provincial and municipal levels were obtained from survey, and 37 policy documents at the national level were obtained from the Peking University's Law Database (www.pkulaw.com). Considering the similarity of contents and the relevance of this study, the national policy documents do not include relevant laws and emergency plans for emergencies other than public health. All the policy documents before 2020 are national (37), covering four areas: emergency management, reserve management, public health event management, and public procurement (Figure 3). A total of 52 policy documents were released and implemented from January to March 2020, focusing on key issues in epidemic procurement management (Figure 4). The documents on supply chain coordination are with the largest number, followed by the procurement and funding guarantee. All policy documents at provincial and municipal levels were issued to meet the needs of emergency procurement management after the outbreak.

Because China's current public procurement law do not regulate emergency procurement, the only document directly related to emergency procurement is the temporary Notification on Procurement Facilitation for Epidemic Prevention and Control. It gives great flexibility for emergency procurement, but there is no detailed guidance (65).

**Figure 5:**
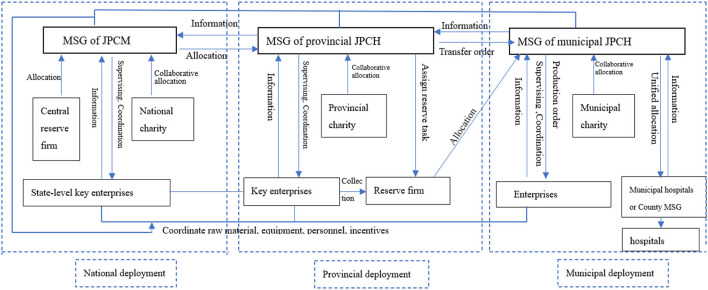
Framework of China's emergency procurement and supply system against COVID-19.

Based on the above data, the authors describe the following framework (Figure 5) of China's emergency procurement and supply system during the major public health event.

## Results

After gathering the data through the research methods of case study and semi-structured interview, this study further used the NVivo12 qualitative analysis software to interpret the collected 14 interview records and 69 policy documents in detail one by one. Based on the dimension of strategic procurement and the corresponding theory and practice, the authors extracted a total of 18 main nodes and 299 reference points. Due to the complementarity in content between the policy documents at different stages, as well as between the policy documents and interview materials, this article combines the two together for text analysis. Both authors were involved in coding the raw data. When there are different opinions, both sides would have an in-depth discussion or consult experts in practice and eventually reach an agreement (66, 67).

In this study, 14 interview records and 69 policy documents collected were interpreted in detail one by one. The NVivo12 qualitative analysis software was used to identify 18 master nodes and 299 reference points corresponding to the strategic procurement dimension. Due to the complementarity between policy documents at different stages and between policy documents and interview data, this article combines them for text analysis. The results of data analysis are shown in Table 4.

**Table 4:** Related nodes and examples.

**Dimension**	**Reference points**	**Main node**	**Reference points**	**Example**
Strategic procurement objectives	30	Resolving public risks	30	“Effectively safeguard the life and health safety of the people and the country's public health security.”(National Policy: GJ-11)
Strategic procurement planning	38	Requirements definition	4	“Relevant departments shall jointly determine and timely adjust the varieties of medicines and medical devices in the central reserve.”(National document: GJ-38)
		Sourcing	4	“Select pharmaceutical reserve enterprises according to their management level, storage conditions, scale and operating efficiency.”(National document: GJ-38)
		Framework agreement	6	“Enterprises undertaking the task of pharmaceutical reserve must sign a pharmaceutical reserve framework agreement with the corresponding pharmaceutical reserve management department” (National document: GJ-38)
		Questions and Suggestions	24	“It is suggested to improve the tiered reserve management system for public health events.” (Interview record: FT-C15)
Status of procurement department	19	Functions of MSG	10	“We will improve the mechanisms for emergency production, procurement, rotation of storage and allocation of emergency supplies, and improve the capacity of comprehensive coordination and classified support for emergency supplies.” (National document: GJ-17)
		Status of MSG	8	“Epidemic prevention and control, medical treatment, scientific research and material support constitute the main parts of epidemic prevention and control mechanism.” (Provincial document: HS-05)
		Function of conventional public procurement department	1	“The public procurement Management Sector of BF in Y City plays the role of procurement consultation. The central public procurement center was not involved.” (Interview record: FT-B12)
Specialization of procurement management	22	Member of MSG	14	“The BIIT is the leader of the MSG because it knows the medical industry better, and the pharmaceutical company is the reserve enterprise.” (Interview record: FT-A12)
		Role of experts in centralized procurement center	8	“Experts from the public centralized procurement center were not involved, and MSG commissioned local pharmaceutical companies to help with the emergency procurement.” (Interview record: FT-B15)
Procurement and supply integration	91	Partnership	8	“There are a total of 16 key producers of emergency supplies in X Province. MSG designated specific personnel to the key enterprises, so as to coordinate production, supervise the unified allocation of emergency supplies.” (Interview record: FT-A12)
		Production process integration	57	“The provincial MSG focused on the production of materials and strive to solve the outstanding problems in key enterprises, such as lack of staff, equipment, materials and logistics.” (Interview record: FT-A11)
		Incentives	26	“Eligible key enterprises will be supported with a no more than one year's interest discount (50% off) of the special loan.” (National document: GJ-18)
Procurement synergy mechanism	99	Synergy between governments	29	“JPCH organized special teams and established responsibilities, coordinated between different levels of government.” (Interview record: FT-A12)
		Synergy between government and market	3	“MSG entrusted three pharmaceutical companies to help collect and store the emergency supplies by paying management fee of 10%.” (Interview record: FT-A12)
		Synergy of regulatory	32	“Market supervisors at all levels should strengthen market law enforcement and intensify efforts to crack down on illegal activities.” (Provincial document: HS-15)
		Synergy of Information	26	“MSG should designate special personnel to be responsible for the statistics and reporting of the information on medical materials and the demand for raw materials every day.” (Provincial document: HS-02)
		Synergy between procurement and donation	9	“All kinds of donated material from home and abroad, except targeted donations, especially those in urgent need, should be allocated by the provincial JPCH.” (Provincial document: HS-04)

## Discussion

### Strategic Procurement Objectives

We find that defusing public risks and maintaining public safety are the core objectives of emergency management from the 12 policy documents on emergency management and reserve management issued before COVID-19. Judging from the documents on public health event management, including COVID-19 epidemic control, safeguarding people's life and health, is the core objective of responding to public health events. As COVID-19 is a major public health emergency, the central government of China required that people's lives and health be given top priority and all-out efforts should be made to prevent and control the epidemic of COVID-19 (68). Putting people at the first place and ensuring people's safety and health is the organizational goal of epidemic prevention and control, as well as the strategic goal of emergency procurement. In this case, the MSG of X Province went all out to organize the production, procurement, and deployment of emergency supplies to ensure the material basis for epidemic prevention and control (interview record: A11).

**Figure 6:**
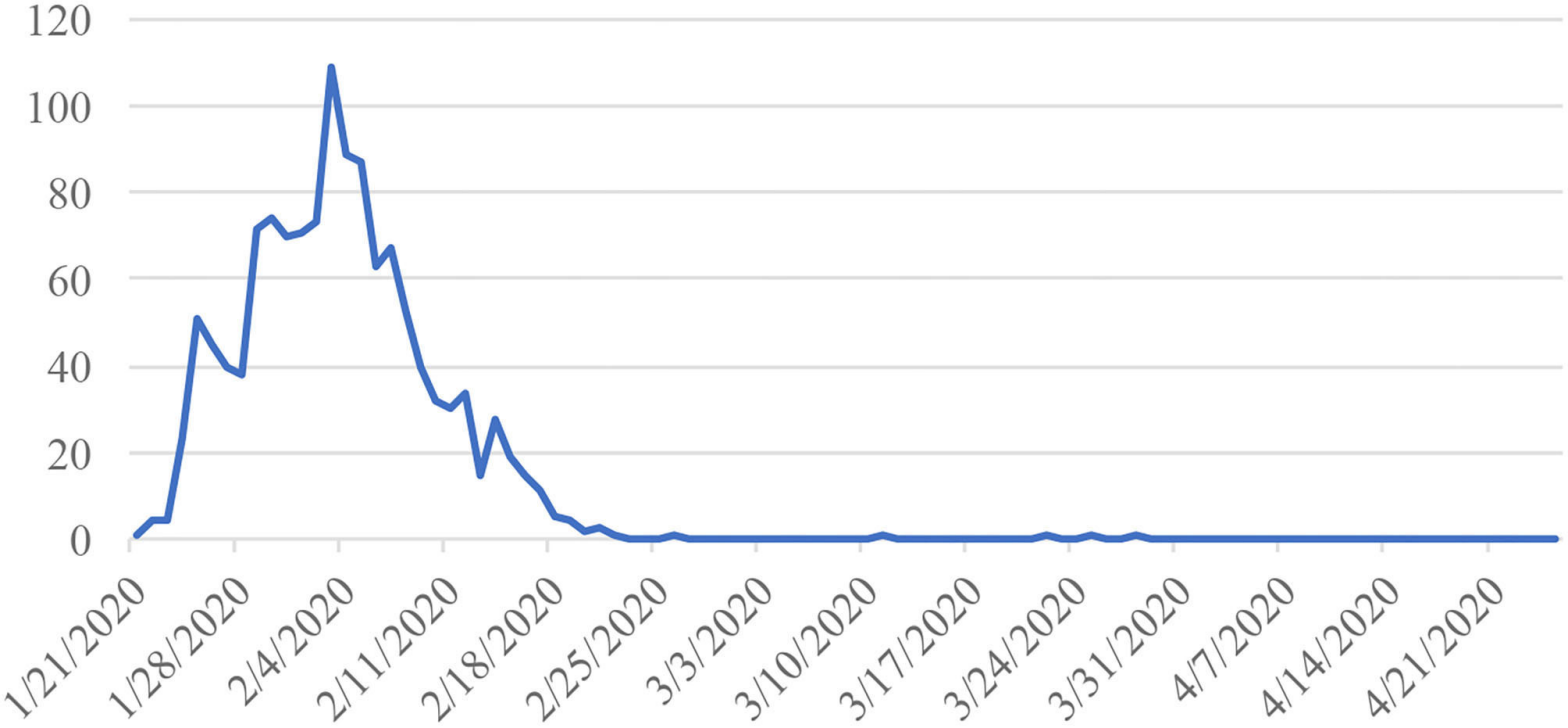
New confirmed cases in X Province.

From the perspective of strategic procurement, public procurement should not only minimize procurement costs but also serve the comprehensive objectives of the public sector (46). China's emergency procurement took the strategic objective of central government as its objective and finally realized the strategic function of protecting people's life and health safety by material support. Figure 6 shows the new confirmed cases from 21 January 2020, to 30 April 2020, which fell to zero at the end of February^2^.

### Strategic Procurement Planning

Demand definition, sourcing, and framework agreement constitute the main contents of strategic planning for emergency procurement, which can be transformed into the issue of strategic reserve in practice. NVivo has identified 14 reference points, 10 of which are from China's emergency plan, emergency system construction plan, and reserve policies. For example, “use pre-signed contracts and other economic means to build a socialized emergency supplies support system” (document: GJ-7), “determine and timely adjust the varieties of drugs and medical devices in central reserve,” and “set up conditions for enterprises undertaking the task of medical reserve” (document: GJ-38). This shows that before the outbreak of COVID-19, China did not lack the top-level design of strategic procurement planning.

However, the survey found that in practice, the procurement planning, such as demand definition and sourcing lags behind, and the framework agreements signed with enterprises in advance are not binding enough. It was only after the outbreak of COVID-19 that relevant departments released the list of key anti-epidemic supplies, began to sort out the emergency medical industry chain, implemented the list-based management of key enterprises, and established cooperative relationships with suppliers. The strategic reserve system established based on procurement and framework agreement has not played its expected role, in fact, resulting in a prominent shortage of reserve of emergency supplies. The reason is that the framework agreements lack effective incentive and constraint mechanism, and the enthusiasm of enterprises participating in the reserve system is insufficient (interview record: A12), which affects the reserve level and the construction of reserve capacity. In the survey, 12 interviewees called for strengthening the strategic procurement planning and strengthening the strategic reserve of emergency supplies.

The reserve of emergency supplies based on strategic procurement planning can buffer emergency demand, shorten procurement cycle, and enhance emergency reaction capacity. The uncertainty of emergencies requires procurement management to take precautions and make strategic planning for emergency procurement (56). The lack of strategic planning in China has become a major problem of emergency procurement management.

### Status of the Procurement Department

In China's response to COVID-19, MSG is the centralized procurement department for emergency supplies. This article analyzes 19 reference points from three aspects: the function and organizational status of MSG and the role of conventional public procurement department. It can be seen that the MSG is responsible for “unified raising, management, and allocation” of emergency supplies and is the department that coordinates procurement, supply, and allocation (National document, GJ-29). During the process of epidemic response, epidemic prevention and control, medical treatment, scientific research, and material support constitute the main functions of JPCH (Provincial document, HS-05). The MSG plays an important role in JPCH. The vice governor of X Province leads the MSG's special production team. The procurement and distribution of emergency supplies reaching a certain value in Y City shall be approved by the deputy mayor. The survey found that the MSG, as a procurement department dealing with COVID-19, was at the top of the emergency response organization.

The strategic function of procurement can be realized only when the procurement department is highly valued by the top management of the organization (37). Therefore, China's emergency procurement department (MSG) has gained the organizational status corresponding to strategic procurement, which provides organizational guarantee for realizing the strategic objectives. However, this study also found that the MSG, as a temporary organization, had no functional connection with the permanent centralized procurement center, resulting in the centralized procurement center almost played no role in the emergency procurement. Therefore, in the context of discussing the normalization of emergency procurement management, the question of how to better coordinate the emergency procurement function with the permanent government procurement function becomes crucial.

### Specialization of Procurement Management

This article extracts 22 reference points from two aspects: the composition of the MSG and the function of the experts in the centralized procurement center. The procurement management team for this epidemic is a cross-sector material support team, involving BIIT, HC, BF, AB, BCA, etc. The BIIT takes advantage of its understanding of the emergency industry and is the leader of the MSG, mainly responsible for supply chain coordination and supplier management. The HC is responsible for gathering emergency needs and assisting in material distribution. The BF is mainly responsible for fund guarantee. The AB is responsible for auditing the distribution of special funds and donated money or goods, material procurement, fund accounting, etc. The Bureau of Market Supervision (BMS) timely supervises and administers the quality, price, and market release of emergency supplies. The Bureau of Transportation (BT) ensures logistics and transportation, and large pharmaceutical companies assist in the collection, storage, distribution, and transportation of emergency supplies. However, in terms of the procurement expert team, experts of centralized procurement center did not participate in the emergency procurement.

Specialization of procurement management is a prerequisite for dealing with the particularity of procurement environment and complexity of procurement management under emergencies and is a key factor affecting the success of strategic procurement (37). The MSG has prominent cross-sector advantages at macrolevel, which can fully make use of the advantages of various departments to better coordinate and guarantee the procurement and supply of emergency supplies. However, the MSG is short of professional force of public procurement on the microlevel. It still needs to replenish procurement experts and let professional people do professional work, so as to make the emergency procurement management more refined and better achieve the strategic objectives of procurement.

### Procurement and Supply Integration

At the beginning of COVID-19, China implemented effective procurement and supply integration measures, including partnership building, production process integration, and incentive measures. The related reference points account for one-third of the total in the text analysis results. In the first 3 months of 2020, 53% of the material support policies were related to procurement and supply integration. Among them, the production process integration is the most prominent. The MSG at all levels makes great efforts to coordinate the resumption of production, supply of raw materials and equipment, and logistics to ensure the smooth flow of the supply chain. In terms of partnerships, the government selected national and provincial key producers of emergency supplies to establish partnerships; set up on-site working groups to assist and supervise the resumption of production and capacity expansion of enterprises; and carried out national, provincial, and municipal unified allocation of emergency supplies. In terms of incentive measures, the governments carry out supporting procurement policies, preferential tax policies, and financial support policies to mobilize the enthusiasm of key enterprises for cooperation. In this case, the integration effect of procurement and supply is remarkable. The company of interviewee D11' only took 3 days to complete the application for production qualification and start the new production line. The daily production capacity of medical protective suits in X Province increased from 4,900 to 40,000 units in 1 month (FT-A11). Table 5 shows the national level daily output growth rate of related products from January, 2020, to April, 2020 (69).

**Table 5:** Production of key medical supplies on April, 2020.

**Type**	**Products**	**Daily capacity**	**Daily output**	**Growth rate** **of daily output**
Protective equipment	Medical protective clothing (10,000 sets)	189	80	90.6 times
Sterilization supplies	Hand sanitizer (ton)	409	308	2.6 times
	84 Disinfectant (10,000 cases)	36.6	11.7	1.6 times
Medical equipment	infrared thermometer (10,000 units)	1.07	0.34	23.3 times
Test items	Virus detection reagent (10,000 units)	1,020	760	58 times

The integration of procurement and supply is an important feature that distinguishes strategic procurement from traditional procurement. Its core is to establish a long-term win-win partnership with suppliers so that suppliers can make greater contributions to the value-added of the organization (60). Because emergency procurement is usually in a market environment of shortage, public procurement management departments need to consider how to organize not only emergency procurement but also supplier relationships (54). China's experiences on procurement and supply integration exactly shows the advantages of strategic procurement.

However, since the MSG is a temporary organization, the partnership between the government and key enterprises is temporary and established by administrative orders, and there is no standard cooperation agreement between the two parties (interview record: A12). Strategic procurement requires the establishment of a long-term stable partnership because long-term partnership is conducive to integrating the partners' advantages and resources, forming innovative solutions. On the other hand, it is also conducive to enhancing the capacity of response to supply chain, consolidating strategic reserve and fundamentally improving the capacity of emergency supporting (31). Therefore, how to combine the temporary mechanism of emergency procurement with the conventional procurement mechanism established by the public procurement law, expand the function of the conventional public procurement system, and realize the integration of procurement and supply under the normal public procurement system are urgent tasks for China.

### Procurement Synergy Mechanism

**Figure 7:**
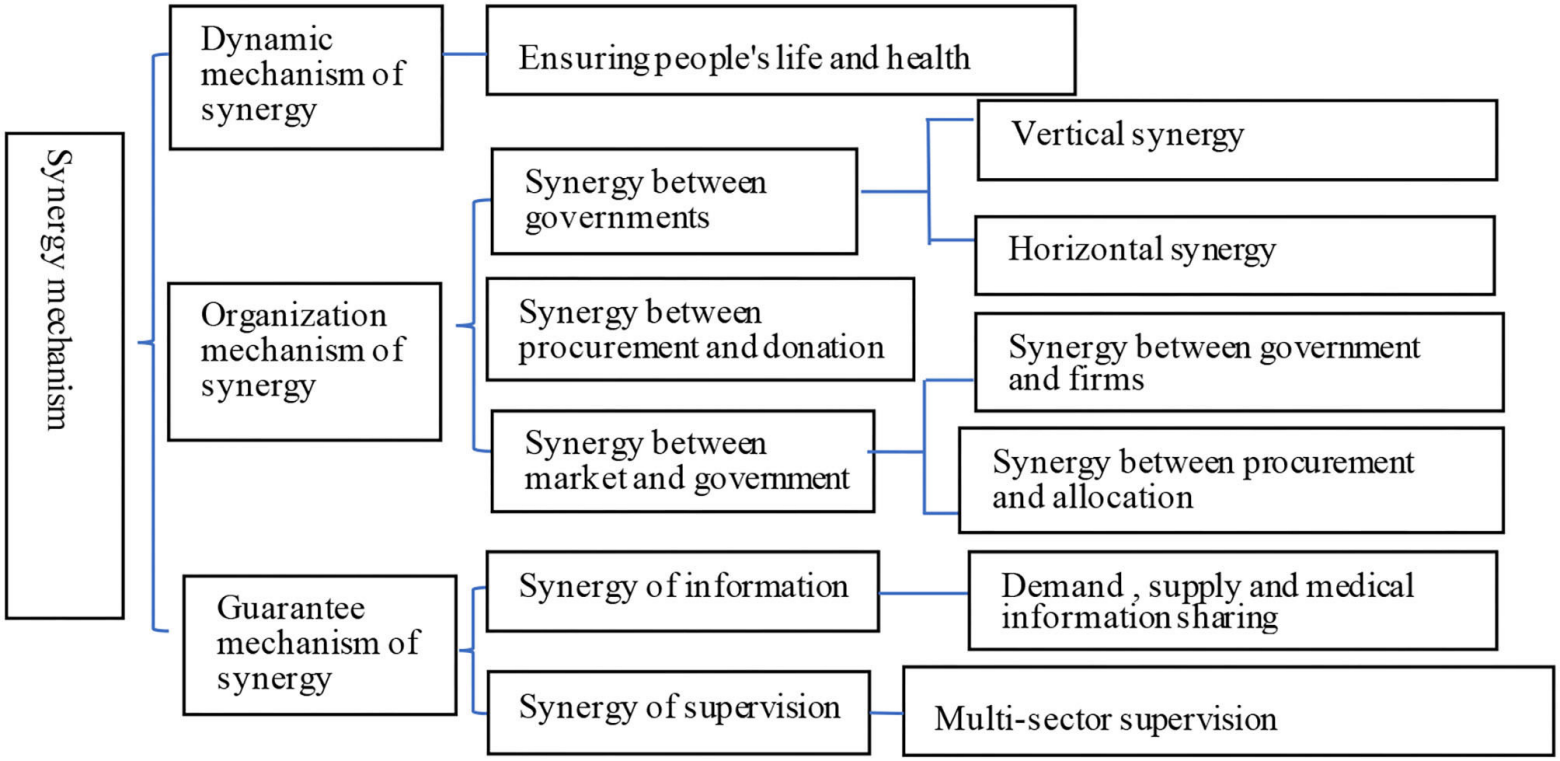
Synergy mechanism of China's emergency procurement.

It is can be seen from the results of NVivo that procurement synergy mechanism is the outstanding feature of China's public procurement to deal with COVID-19. The reference points of it rank first among all the main nodes. Figure 7 shows the detailed procurement synergy mechanism in China's procurement measures in response to COVID-19. The strategic goal of protecting people's life and health constitutes the dynamic mechanism of synergy. The synergy between governments, the synergy between government and market, and the synergy between procurement and donation constitute the organizational mechanism of synergy, and the synergy of information and supervision constitutes the guarantee mechanism of coordination. One aspect of synergy between governments is vertical synergy between different levels of governments, such as the MSG of State JPCH coordinates the dispatching of raw materials and production equipment for key enterprises in X Province. The other aspect is horizontal synergy between the same levels of governments, such as members of MSG in X Province and Y City work together to coordinate the production, procurement, and deployment of emergency supplies. The synergy between government and market includes the partnership between government and key producers, the synergy among supply, centralized procurement, and unified allocation through the nation, province, and city. The synergy between procurement and donation lies in that all donated funds and materials are distributed in coordination with the JPCH. The synergy of information requires the key production enterprises and designated medical institutions gather supply and demand information of emergency supplies, then build up an information system together with the epidemic information. The synergy of supervision between different sectors ensures the production quality and reasonable price of products and ensures the efficiency of material allocation and fund use.

The procurement synergy mechanism is the most important guarantee for the implementation of strategic procurement in China during the outbreak of COVIV-19. Since emergency procurement involves a large number of stakeholders, the synergy mechanism facilitates mobilizing different resources within a unified system (62), improves the efficiency of procurement management (70), and achieves organizational strategic objectives.

## Conclusion

From the perspective of strategic procurement, China's experience on public procurement in responding to COVID-19 lies on emphasizing and realizing the strategic function of emergency procurement. Specifically, first, as a strategic procurement department, the MSG has established the strategic goal of safeguarding people's life and health. Second, the MSG is at the top of the emergency response organization (JPCH), which ensures the implementation of strategic procurement objectives. Third, a cross-sector procurement team leverages the strengths of different administrative departments to ensure the production, procurement, and allocation of emergency supplies. Fourth, strong procurement and supply integration, including highlighting production process integration, maximize the emergency materials supply and successfully reconcile the extreme market shortage. Fifth, a comprehensive and in-depth procurement synergy mechanism fully mobilizes the resources and capabilities of the public sector, the private sector, and charitable organizations, and serve the strategic goal of safeguarding people's life and health.

However, to meet the requirements of strategic procurement, the realization degree of strategic procurement in China's emergency situation still needs to be improved. First, a strategic planning for emergency procurement is not fully implemented. As a result, reserves are seriously insufficient, emergency supplies rely on temporary supply, and emergency production depends on temporary mobilization. In particular, the reserve mechanisms needed for emergency procurement, such as partnerships and framework agreements, are not well prepared. Second, the emergency procurement department (MSG) is a temporary team, and emergency procurement relies on temporary policies, which lacks a standing mechanism. The procurement and supply function of the MSG has not been combined with that of the centralized procurement center, and the procurement management team has not absorbed public procurement experts, resulting in the lack of specialization of procurement management. Thirdly, the conventional public procurement system fails under the shortage market, so it needs to be improved and better integrate procurement and supply.

The crux of the problems mentioned above results from the insufficient supply of the emergency procurement institution. This study suggests that the public procurement law should be reconstructed to accommodate the emergency procurement with greater institutional flexibility and promote the transformation of the public procurement to strategic procurement.

### Making Public Procurement Law Compatible With Emergency Procurement

The emergency procurement system should not exist independently of the conventional public procurement system. On the contrary, emergencies of different harm degrees, at different stages, along with different degrees of urgency and different situations of market supply and demand of emergency supplies, require different procurement methods and procedures, and adapt different procurement strategies. The public procurement law should also include all procurement situations for the purchaser to choose. Excluding emergency procurement from the scope of application of the procurement law, although it can give temporary maximum effectiveness, does not contribute to the establishment of a more flexible, scientific, and compatible public procurement system. China's public procurement legal system should include the emergency procurement situation and be suitable for different types of emergencies and dynamic changes in the process of emergency. Public procurement law compatible with emergency procurement may avoid the marginalization of centralized procurement centers and improving the level of specialization in emergency procurement management, as emergency procurement will be the conventional function of the centralized procurement center. Public procurement law compatible with emergency situation is also a common feature of major public procurement laws in the world (58, 59, 63, 71).

### Promoting the Transformation of Public Procurement to Strategic Procurement

Firstly, the government should better integrate national strategic objectives into public procurement objectives. Procurement in response to the COVID-19 pandemic highlights that strategic procurement should be more conducive to achieving the government's strategic objectives. This good practice can be extended to the entire public procurement system to promote more economic and social strategic goals, which is also part of the value for money pursuit of public procurement.

Secondly, strategic procurement planning should be taken as an important part of the emergency procurement management system, and the value of the framework agreement for emergency procurement and reserve should be brought into full play. In fact, a framework agreement is the basis of partnership. Applying a framework agreement to build better reserve is also widely adopted in the world (55).

Finally, procurement and supply integration should be improved. China's current public procurement law ignores the supply management function of procurement, resulting in its failure in response to COVID-19. Therefore, to better play the strategic function of procurement, we should attach importance to the supply management function of procurement. Further, the government should manage the suppliers from compliance to the value they can create, from temporary short-term cooperation to emphasizing a long-term partnership, so that suppliers can truly become partners of the government in public management. Italy, the Republic of Korea, and the UK have all successfully integrated emergency procurement with supply management during the outbreak of COVID-19 (36).

**Figure 8:**
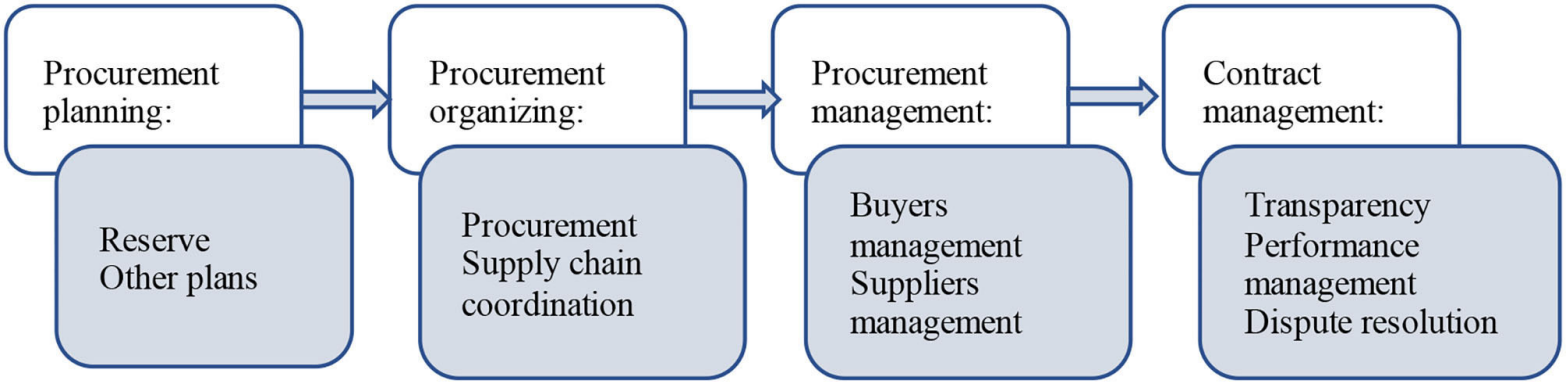
Public procurement system.

In summary, the desirable public procurement system is shown in Figure 8.

### Study Limitations

Due to the social distancing policies during the COVID-19 pandemic, the number of people interviewed for this study was limited. In addition, China does not require information disclosure for emergency procurement, so it is impossible to obtain accurate information on supply and demand of emergency medical supplies, resulting in limited data. As a major emergency, COVID-19 has not been defeated and further follow-up research on procurement against it is still needed.

## Data Availability Statement

The raw data supporting the conclusions of this article will be openly available on request.

## Ethics Statement

The Central University of Finance and Economics, China, approved this study. All participants provided informed consent prior to study participation.

## Author Contributions

FC has supervised this study that was carried out by RY. RY collected and analyzed the data. Both FC and RY reviewed and modified the article and approved the submitted version.

## Funding

The authors acknowledge financial support from the National Social Science Foundation of China (15ZDB174). This paper is also part of results of Guizhou Philosophy and Social Science Program (20GZQN19).

## Conflict of Interest

The authors declare that the research was conducted in the absence of any commercial or financial relationships that could be construed as a potential conflict of interest.

## Publisher's Note

All claims expressed in this article are solely those of the authors and do not necessarily represent those of their affiliated organizations, or those of the publisher, the editors and the reviewers. Any product that may be evaluated in this article, or claim that may be made by its manufacturer, is not guaranteed or endorsed by the publisher.

## Footnotes

^1^Available online at: https://voice.baidu.com/act/newpneumonia/newpneumonia (accessed November 22, 2020).

^2^Available online at: https://www.gtarsc.com (accessed November 22, 2020).
